# Genomics and integrative clinical data machine learning scoring model to ascertain likely Lynch syndrome patients

**DOI:** 10.1038/s44276-025-00140-7

**Published:** 2025-05-05

**Authors:** Ramadhani Chambuso, Takudzwa Nyasha Musarurwa, Alessandro Pietro Aldera, Armin Deffur, Hayli Geffen, Douglas Perkins, Raj Ramesar

**Affiliations:** 1 Department of Global Health and Population, Harvard T. Chan School of Public Health, Boston, MA USA; 2 UCT/MRC Genomics and Precision Medicine Research Unit, Division of Human Genetics, Department of Pathology, University of Cape Town, Cape Town, South Africa; 3 IndigenAfrica, Inc., Cape Town, South Africa; 4 Department of Public Health and Bioinformatics, University of Cape Town, Cape Town, South Africa; 5 Department of Global Health, School of Medicine, University of New Mexico, Albuquerque, NM USA; 6 Institute of Infectious Disease and Molecular Medicine, University of Cape Town and Affiliated Hospitals, Cape Town, South Africa

## Abstract

**Background:**

Lynch syndrome (LS) screening methods include multistep molecular somatic tumor testing to distinguish likely-LS patients from sporadic cases, which can be costly and complex. Also, direct germline testing for LS for every diagnosed solid cancer patient is a challenge in resource limited settings. We developed a unique machine learning scoring model to ascertain likely-LS cases from a cohort of colorectal cancer (CRC) patients.

**Methods:**

We used CRC patients from the cBioPortal database (TCGA studies) with complete clinicopathologic and somatic genomics data. We determined the rate of pathogenic/likely pathogenic variants in five (5) LS genes (*MLH1, MSH2, MSH6, PMS2, EPCAM*), and the *BRAF* mutations using a pre-designed bioinformatic annotation pipeline. Annovar, Intervar, Variant Effect Predictor (VEP), and OncoKB software tools were used to functionally annotate and interpret somatic variants detected. The OncoKB precision oncology knowledge base was used to provide information on the effects of the identified variants. We scored the clinicopathologic and somatic genomics data automatically using a machine learning model to discriminate between likely-LS and sporadic CRC cases. The training and testing datasets comprised of 80% and 20% of the total CRC patients, respectively. Group regularisation methods in combination with 10-fold cross-validation were performed for feature selection on the training data.

**Results:**

Out of 4800 CRC patients frorm the TCGA datasets with clinicopathological and somatic genomics data, we ascertained 524 patients with complete data. The scoring model using both clinicopathological and genetic characteristics for likely-LS showed a sensitivity and specificity of 100%, and both had the maximum accuracy, area under the curve (AUC) and AUC for precision-recall (AUCPR) of 1. In a similar analysis, the training and testing models that only relied on clinical or pathological characteristics had a sensitivity of 0.88 and 0.50, specificity of 0.55 and 0.51, accuracy of 0.58 and 0.51, AUC of 0.74 and 0.61, and AUCPR of 0.21 and 0.19, respectively.

**Conclusions:**

Simultaneous scoring of LS clinicopathological and somatic genomics data can improve prediction and ascertainment for likely-LS from all CRC cases. This approach can increase accuracy while reducing the reliance on expensive direct germline testing for all CRC patients, making LS screening more accessible and cost-effective, especially in resource-limited settings.

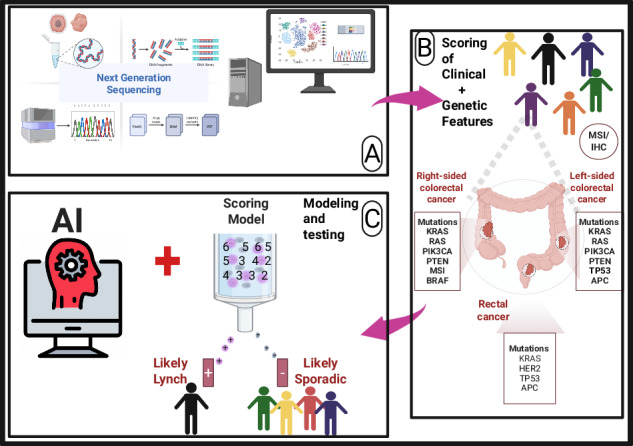

## Introduction

Lynch syndrome (LS) is the most common hereditary colorectal cancer (CRC) syndrome worldwide [1, 2]. It is caused by inherited germline pathogenic variants (GPV) in one of the four DNA mismatch repair (MMR) genes: *MLH1, MSH2, MSH6, PMS2* or the *EPCAM* gene [3, 4]. LS accounts for 2-3% of all diagnosed CRC patients and it is often under-recognized. Confirmation of LS diagnosis requires genetic germline laboratory testing, which can be prohibitively expensive [3, 5, 6]. Despite its high prevalence as early-onset CRC and a significant public health impact, screening for LS remains a challenge due to the complexity of the current molecular diagnostic algorithms and the cost associated with specialized molecular diagnostics [3, 7]. In resource-limited healthcare settings, these challenges are further amplified [8–10]. This is because there are limited accurate, cost-effective, and adaptable methods such as machine learning-based models that can simultaneously integrate statistics, comprehensive clinical and somatic genomic features to ascertain and predict likely-LS cases in cohorts of CRC patients [11–14]. By addressing this pressing need, using a novel approach that leverages machine learning techniques, we can improve LS screening in resource limited settings [15, 16].

We previously innovated a scoring model to screen for LS in CRC patients using basic clinical and molecular genetic features and validated it successfully in our genetic clinics [8, 15, 17]. We aim to improve this scoring model by involving statistics and a machine learning approach [12, 16]. Previously, several efforts have been made to develop prediction models for LS screening and prevention [18]. Some of the notable ones include the Amsterdam criteria I and II, and the revised Bethesda guidelines, which utilize clinical and family history information to identify patients at risk for LS [19, 20]. However, these models are limited by their reliance on detailed and accurate family history, which may not always be available or reliable [21, 22]. Additionally, these models do not incorporate the wealth of somatic genomic data that has become increasingly clinically available in recent years [21]. Another notable LS prediction model, PREMM5, uses personal and family history of cancer to predict the likelihood of identifying a GPV in one of the five LS-associated genes [23, 24]. While this model incorporates genetic information, it does not take into account the full range of somatic genomic/molecular features associated with LS, such as microsatellite instability (MSI) [24]. Furthermore, all these models were developed and validated in high-income countries with advanced healthcare infrastructure [13, 24, 25]. Their performance in resource-limited settings, where access to germline genetic testing and specialized molecular diagnostics is often limited, remains untested [23, 26]. The PREMM Plus model, an extension of the PREMM5 model, is another significant contribution in the field of LS prediction models. It incorporates personal and family history of cancer to predict the likelihood of detecting a pathogenic variant in one of the five LS-associated genes and it was expanded to include other hereditary cancer genes (e.g., *APC, TP53, MUTYH, CHEK2*) [14]. However, despite these advancements, the PREMM Plus model still has challenges that limit its effectiveness. For example, it does not include somatic molecular markers such as immunohistochemistry (IHC) staining results of tumor tissues to provide a more comprehensive prediction model from retrospective CRC data [14]. Also, it relies heavily on the availability and accuracy of detailed personal and family cancer history, which may not always be accessible or reliable, especially in resource limited settings [14, 26].

When hereditary mutations pair with a random pathogenic somatic mutation in the ‘normal’ allele of a mismatch repair gene, it results in a deficiency in the DNA mismatch repair mechanism [27–29]. This deficiency leads to an accumulation of length-altering mutations at microsatellites, a phenomenon known as MSI [29, 30]. However, more critically, there is a decreased susceptibility to apoptosis triggered by DNA damage recognized by the MMR pathway [27, 31]. This characteristic provides a potent selectable Darwinian advantage to such cells [27]. When the MMR system is defective due to mutations in MMR genes or epigenetic silencing of *MLH1*, it leads to MSI, which is characterized by an increased rate of mutations within microsatellites, which are short repetitive DNA sequences- predisposing individuals to cancer [31, 32]. Although not specific, clinically, LS is associated with early-onset CRC, tumor right sidedness, high tumor infiltrating lymphocytes (TILs), and a positive familial burden of cancer [15, 27, 33, 34].

We hypothesize that integration of a comprehensive simultaneous scoring of these clinical and somatic genomic features specific to LS and the involvement of a statistical machine learning framework, could provide a more precise prediction of likely-LS cases from a cohort of CRC patients in a limited resource setting [12, 15, 35–37]. By leveraging machine learning techniques, we can efficiently handle large amounts of data and identify complex patterns that may not be apparent with traditional methods [12, 35, 37, 38]. This could potentially complement existing models and lead to a more accurate and cost-effective prediction approach for LS in CRC patients [37, 39, 40].

To address our hypothesis, we used the cBioPortal database (TCGA CRC studies) to develop a unique machine learning scoring model that can effectively discriminate likely-LS patients and sporadic cases from a cohort of CRC patients.

## Materials and methods

### Patient selection

The cBioPortal database for cancer genomics is a publicly available database that consists of data from thousands of cancer patients sequenced at the time of diagnosis with clinical and somatic genomic data after tumor testing. This includes CRC clinical and genomic features which can be used to develop a machine learning model to screen for likely-LS cases using these features [41, 42]. Data from all patients’ diagnosed with CRC from the TCGA studies were downloaded, and analyzed. Exclusion criteria were applied to specify CRC patients with missing data for genomics analysis. These criteria resulted in 524 patients with complete clinical and tumor sequencing data from 11 TCGA studies.

### Sample size estimation

The sample size comprised 80% (*n* = 420) of the total observations (*n* = 524) which were allocated to the training dataset, and the remaining 20% (*n* = 104) were used for testing. While we did not use any formal statistical methods to calculate this sample size, the data were stratified based on the outcome of likely-LS patients to preserve the original distribution in the training and testing datasets. This approach ensures that the convenient sample reflects the population characteristics, which indirectly supports adequate power for detecting the effect size.

### Inclusion criteria

i. Complete clinicopathological data.
ii. Somatic genomics data available.
iii. Data for key variables such as tumor stage, MSI status, and genetic mutations.

### Exclusion criteria

Patients with incomplete data (e.g., missing clinicopathological or genomic information) were excluded from the analysis.

### Clinical and somatic variant annotation methods

**Fig. 1:**
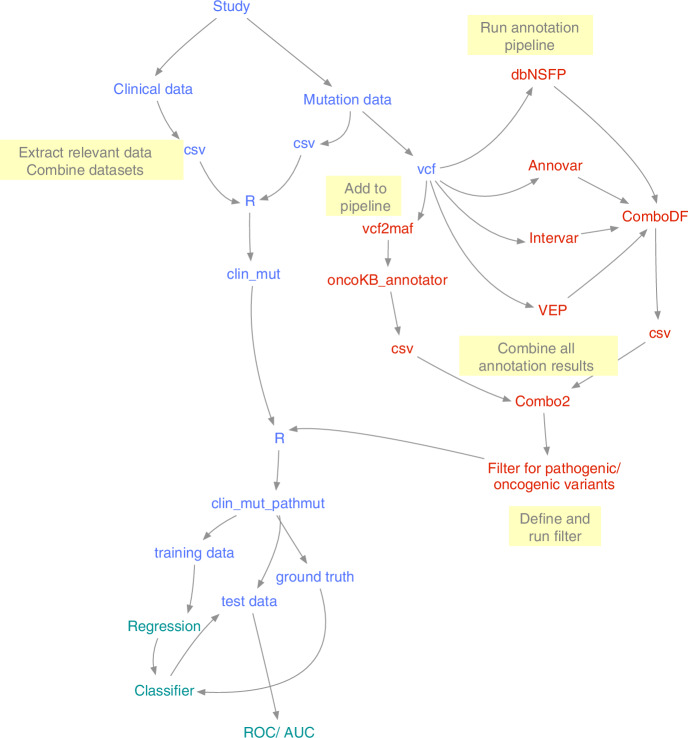
An illustration of clinical, mutation data and somatic variant annotation methods for an overall approach and the pre-designed bioinformatic annotation pipeline. Annotation was performed only for molecular data by running annotation pipeline from various online databases such as dbNSFP, Annovar, Intervar, VEP, and oncoKB. Clinicopathologic data and somatic mutation data were analyzed separately and then combined in a training and testing data for machine learning modelling.

We ascertained CRC patients from the TCGA studies with clinicopathologic and somatic mutation data. Clinical data encompassed demographic variables such as age, sex, ethnicity, and health-related information including family history, and clinical outcomes. The rate of pathogenic/likely pathogenic variants in 5 LS genes (*MLH1, MSH2, MSH6, PMS2, EPCAM*) and the *BRAF* mutations were determined using a pre-designed bioinformatic annotation pipeline (Fig. 1). Annovar 2023 update (https://wannovar.wglab.org/, last visited on 02/03/2024),

Intervar 2022 update (https://wintervar.wglab.org/, last visited on 03/04/2024), Variant Effect Predictor (VEP) 2023 update, https://ensembl.org/info/docs/tools/vep/index.html, last visited on 04/05/2024), and OncoKB 2022 update (https://www.oncokb.org/, last visited on 27/06/2024) software tools were used to functionally annotate and interpret sequenced somatic variants of [43–47]. The OncoKB precision oncology knowledgebase was used to provide information on the effects of the identified variants [47]. In parallel, mutation data were derived from high-throughput sequencing assays in the cBioPortal database, which identified genomic variants across the patients’ genomes [48, 49]. To ensure the integrity of the research, only the most pertinent variables were selected for inclusion in the combined dataset. This selection process was guided by a set of predefined criteria that focused on relevance to the research at hand, the quality and completeness of the data, and the potential for clinical significance of the genetic variants. Once selected, the data underwent a stringent pre-processing phase, where it was normalized to ensure consistency across the dataset. This involved standardizing measurement units, coding categorical variables, and addressing missing or outlier values through accepted statistical methods [50]. The fusion of two rich datasets in comma-separated values (csv) files for clinical data and mutation data into a single, cohesive dataset was carried out using advanced data integration techniques (Fig. 1). This merged dataset was designed to facilitate the cross-referencing of clinical characteristics with genomic mutation data, enabling a multifaceted analysis of the interplay between genetic factors and clinical manifestations of LS.

All somatic variants were found in the sequencing data using FFPE tumor samples. The biological effect of all variants were fully annotated using the Oncomine proprietary software (and further explored with public databases such as onco-KB, Annovar, Intervar, Ensembl Variant Effect Predictor (VEP), and dbNSFP in order to confirm the pathogenicity of variants) using available guidelines [47, 51, 52]. In terms of the latter workflow: genomic variants were exported as variant call files (VCFs) by manual construction. The VCFs were converted to mutation annotation format (MAF) files (vcf2maf includes VEP annotations) and annotated using onco-KB API. The results were integrated from annotation to specimen/individuals with oncogenically-relevant variants to review and reveal the histopathology. An example of a customized workflow being used in our analysis is shown in Fig. 1.

To enhance the mutation data with deep biological insights, an advanced annotation pipeline was employed. This pipeline was designed not just to annotate but to ascribe a predictive value to the genomic variants, enabling a more nuanced understanding of their potential clinical significance. The mutation data collected were subjected to a rigorous and multifaceted annotation pipeline, leveraging an array of state-of-the-art bioinformatics tools to enrich the raw sequencing data with valuable biological context and functional insights [53, 54].

### Functional prediction with dbNSFP

The dbNSFP was the first layer of our annotation process, which provided a holistic view of the functional impact of non-synonymous SNPs. We employed dbNSFP, a comprehensive compendium that consolidates a variety of functional predictions and annotations for human non-synonymous SNPs, as well as splice site variants using vcf files. The use of dbNSFP allowed us to access predictive information from multiple algorithms concurrently, which includes SIFT, PolyPhen-2, and MutationTaster, among others [55]. This provided a multifaceted view of the potential impact of each variant on protein function, stability, and interaction, enriching the dataset with a nuanced perspective on variant deleteriousness. Each variant was subjected to a battery of in silico predictive models incorporated within dbNSFP [56]. The integration of these predictions allowed us to prioritize variants based on their potential to affect protein function and, by extension, their likelihood of contributing to the LS phenotypes.

### Genome-wide annotation with Annovar

With Annovar, we applied a genome-centric approach to annotation. By mapping variants to specific genomic coordinates and regions, we were able to determine their locational relevance in relation to gene structures and regulatory elements [44]. Annovar facilitated the enrichment of our dataset with information on variant occurrence within specific genomic features such as coding sequences, introns, promoter regions, and untranslated regions (UTRs), which provided a scaffold for interpreting the potential impact of these variants at the molecular and cellular levels [57]. Annovar was utilized to integrate information from a plethora of genomic databases. It mapped each variant to genomic regions, such as exons, introns, and intergenic regions, and annotated them based on gene function, allowing us to understand the broader genomic context of the mutations.

### Clinical interpretation with Intervar

The clinical significance of each variant was assessed using Intervar, which adjudicated the pathogenicity based on established American College of Medical Genetics and Genomics/Association for Molecular Pathology (ACMG/AMP) criteria [58]. This tool synthesized data from multiple sources, including population frequency databases, *in silico* predictive scores, and published literature on mutational effects, to assign standardized pathogenicity scores. By adopting the ACMG/AMP standards, we ensured that our clinical interpretation aligned with globally recognized guidelines, bolstering the clinical relevance of our findings. Intervar, aligned with the ACMG/AMP 2015 guidelines, was instrumental in the clinical interpretation of variants, providing classifications ranging from benign to pathogenic based on a well-established framework [59]. This step was critical for the clinical validation of the variants, ensuring that the findings were grounded in a clinically relevant and standardized interpretative system [60].

### Data integration and filtration for clinical relevance

Once the mutation and clinical data were unified, the combined dataset underwent rigorous quality assurance processes. These processes included data reconciliation, where we ensured congruence between the clinical phenotype and genomic data, a crucial step in maintaining the integrity of our research. To further streamline the dataset for high-level analytical processes, we implemented a sophisticated filtration algorithm. This algorithm was designed to parse through the integrated dataset, identifying and retaining only those variants with established pathogenicity or oncogenic potential. The criteria for this selection were informed by the latest findings in the field, including emerging data on variant-pathogenicity associations and evolving treatment protocols [61].

### Scoring and regression modelling

We scored the clinicopathologic and mutation data automatically using a designed machine learning model to discriminate between likely-LS and not-LS (sporadic) CRC cases. The training and testing datasets comprised of randomly selected 80% and 20% of the total CRC patients, respectively [14]. Leveraging the training set, a sophisticated regression model was developed to discern intricate relationships among the variables. This model was constructed to capture the complex interplay between genomic variants and their clinical manifestations, employing advanced statistical techniques to model these relationships accurately. The choice of the logistic regression model was dictated by the nature of the data and the specific research questions at hand. The development of this regression model was an iterative process, involving the tuning of parameters and the selection of features based on their statistical significance and clinical relevance. This careful calibration ensured that the model was both robust and interpretable, providing a solid foundation upon which the predictive classifier was built [62, 63].

With the regression model as its backbone, the predictive classifier was then constructed. This classifier was designed to leverage the insights of the regression model to predict the pathogenicity of variants in the test set similar to published studies [64–67]. The development of the classifier was an iterative process, involving rounds of training, testing, and refinement to optimize its performance. Machine learning techniques (decision trees, random forests, and support vector machines) were evaluated for their suitability in this context [68, 69]. The final choice of algorithm was informed by its performance in preliminary tests and its ability to handle the high-dimensional, heterogeneous nature of the genomic data [70]. Techniques such as ensemble learning, including Random Forests and Gradient Boosting Machines, were explored for their ability to handle complex, non-linear relationships within the dataset [71, 72]. These methods combined multiple weak learners to form a strong predictive model, effectively reducing variance and bias, and improving prediction accuracy. To augment the classifier’s predictive capability, integration of multimodal data sources was considered [73, 74].

A logistic regression formula for the model was developed to quantify the probability of likely-LS by incorporating a diverse array of predictors, including demographic information, tumor characteristics, and critical genetic mutations associated with LS. Each predictor was assigned a unique coefficient, reflecting its contribution to the overall risk of likely-LS. Central to the design of the model is the integration of genetic markers, mutations in *MLH1*, *MSH2*, *MSH6*, *PMS2*, and *EPCAM* genes that provided a nuanced assessment of LS likelihood. This genetic component is pivotal, as these mutations are instrumental in the pathogenesis of LS and thus serve as robust indicators of the syndrome [75, 76].

### Statistical screening formula for clinical, MSI and genetic risk score

The formula results demonstrated the predictive power of the model, encapsulated in the logistic function, which transforms the calculated log odds into a probability measure. This transformation is crucial for clinical interpretation, providing a direct estimation of LS risk that can inform subsequent clinical decisions. The probability output ranges between 0 and 1, where values closer to 1 indicate a higher likelihood of LS. This formula has already been incorporated in our online LS screening web tool for our model.

The model equation is written as:

$$\log \left(\frac{{\hat{p}}_{{\rm{Lynch}}{like}}}{1-{\hat{p}}_{{\rm{Lynch}}{like}}}\right)=-7.1636672761$$

+ 0.1715238837 × (age > 60)

+ 0.0027460644 × (tumour stage = 1-2)

+ 0.1947251040 × (tumour stage = unknown)s

+ 0.0003628616 × (sex - male)

+ 0.2756984988 × (history other malignancy = yes)

+ 0.0817041000 × (history other malignancy = unknown)

+ 0.0229552787 × (history colon polyps = unknown)

+ 0.2533830558 × (Family history colorectal cancer = 1-2)

+ 0.1592159679 × (Family history colorectal cancer = unknown)

+ 0.1723759512 × (TLI lymph = unknown)

+ 0.2016103139 × (Tumour side = right-sided)

+ 0.0343689618 × (Tumour side = unknown)

+ 1.0887288363 × (MSI status = MSI)

+ 0.0731597893 × (BRAF = positive)

+ 9.2051175359 × (MSH2 = positive)

+ 9.3988266557 × (PMS2 = positive)

+ 0.7632086747 × (EPCAM = positive)

+ 9.3834143609 × (MSH6 = positive)

- Let us call the above equation on the right-hand side **X**
- After calculating the log-odds, to obtain the probability of likely-Lynch from the model, a logistic function below must be used:
  $${\hat{p}}_{{\rm{Lynch}}\, {like}}=\frac{{e}^{X}}{1+{e}^{X}}$$
  Where X is the sum of the log-odds. This function ensures the predicted probability is between 0 and 1.
  This breakdown shows how each factor contributes to the likelihood of having “Likely-Lynch” characteristics and helps interpret the logistic regression model more clearly.
- To obtain the clinical score points for each risk factor a transformation is applied to the model coefficient for each risk factor: (coefficient*10/315.0572) X100. Because software for all the computations was used, the rounding off the values might differ slightly.

Note: This rounding note suggests that due to the use of software for computations, the exact values may slightly differ when calculated manually or with different software (due to rounding off decimal places. This risk scoring approach is essential for identifying individuals at high risk of being likely-LS, enabling targeted genetic testing and surveillance strategies. It embodies the precision medicine ethos by leveraging individual patient data to inform clinical decision-making.

This logistic regression model predicts the probability of a CRC patient to be “Likely-LS” as follows:

- **Log-Odds calculation**: The log-odds of having “Likely-LS” characteristics (P^Lynch like) is computed from various factors, each with an associated coefficient.
- **Coefficients**: Each term’s coefficient represents the impact of that factor on the log-odds. A positive coefficient increases the log-odds, while a negative one decreases it.

**Factors in the model are as follows:**

1. Age ( ≥ 60): Being 60 years or older contributes positively to the log-odds.
2. Tumor stage (1-2): Early-stage tumors (stages 1-2) slightly increase the log-odds.
3. Tumour stage (Unknown): Unknown tumor stages have a significant positive impact.
4. Sex (Male): Being male has a very small positive effect.
5. History of other malignancies (Yes): A history of other cancers increases the log-odds.
6. History of other Malignancies (Unknown): Unknown history also adds positively.
7. History of colon polyps (Unknown): Unknown history of colon polyps adds a small positive value.
8. Family History of colorectal cancer (1-2): A family history (1-2 relatives) increases the log-odds.
9. Family History of Colorectal Cancer (Unknown): Unknown family history contributes positively.
10. TLI Lymph (Unknown): Unknown TLI lymph status adds to the log-odds.
11. Tumour Side (Right-Sided): Right-sided tumors significantly increase the log-odds.
12. Tumour Side (Unknown): Unknown tumor side slightly increases the log-odds.
13. MSI Status (MSI-H): Having high microsatellite instability (MSI-H) is a major positive contributor.
14. *BRAF* (Positive): Positive *BRAF* status adds a small positive value.
15. *MSH2* (Positive): Positive *MSH2* status has a very large positive impact.
16. *PMS2* (Positive): Positive *PMS2* status also has a very large positive impact.
17. *EPCAM* (Positive): Positive *EPCAM* status significantly increases the log-odds.
18. *MSH6* (Positive): Positive *MSH6* status has a very large positive impact.

### Statistical analysis

We used R programming (version 4.4.4), renowned for its extensive libraries and capabilities in statistical computing and graphical representation. The validation of the performance of the classifier was conducted through rigorous statistical analyses, with a particular focus on Receiver Operating Characteristic (ROC) and Area Under the Curve (AUC) metrics. The ROC curve, a plot of the true positive rate against the false positive rate at various threshold settings, provided a comprehensive view of the discriminatory capability of the classifier. The AUC metric, derived from the ROC curve, offered a single, consolidated measure of the overall performance of the classifier, with values closer to 1 indicating superior predictive accuracy. These analyses were critical in assessing the efficacy of the classifier in distinguishing between likely-LS and non-LS, providing a quantifiable measure of its performance. The insights gained from ROC and AUC analyses informed further refinements to the classifier, ensuring its robustness and reliability in predicting likely-LS.

The foundation of our analysis was a sophisticated regression framework, designed to meticulously identify and evaluate significant predictors of likely-LS. We employed a variety of logistic regression models for binary outcomes, supplemented by more complex models like ridge, LASSO, and elastic net regression for scenarios with high-dimensionality and multicollinearity. These models were instrumental in discerning the intricate relationships between genetic variants and their clinical manifestations, with variable selection techniques applied to isolate the most informative predictors.

In addition to ROC and AUC, Precision-Recall curves were plotted, especially given the imbalanced nature of the dataset where likely-LS might be much rarer than non-likely LS (Sporadic). The F1 score, a harmonic mean of precision and recall, was also calculated to provide a single metric that balances both the false positives and false negatives, offering a nuanced view of the classifier’s performance in scenarios where class imbalance is significant. Sensitivity analyses were conducted to assess the robustness of the performance of the classifier to variations in the dataset and model parameters. This involved systematically varying key parameters and observing the impact on model performance, thereby ensuring that the predictive capabilities of the classifier were stable and reliable under different conditions.

A logistic regression model was used to estimate the probability of a patient being likely-LS, In the logistic regression formula, the log odds of the patient likely to have LS were calculated as a linear combination of various predictor variables, which include patient age, tumor stage, gender, and family history, among others, each weighted by a regression coefficient. These coefficients quantify the influence of each predictor on the likelihood of LS. The logistic function (the inverse logit function) was then applied to these log odds to obtain the probability (ranging between 0 and 1) of a patient having LS. Group elastic net in combination with 10-fold cross-validation to select the hyperparameters for feature selection was used. α (the regularization penalty) = 0.03 was determined from 10-fold cross-validation and λ (the shrinkage penalty) = 0.00546983 was determined from 10-fold cross-validation.

## Results

### Patients cohort and demographics

The demographic and clinical characteristics of the cohort comprising 524 CRC patients, including 40 who are likely-LS and 484 who are sporadic (not-LS). Notably, the likely-LS cohort exhibits a significantly higher percentage (70%, *p* = 0.0311) of early tumor stage (Stage I-II) and CRC located on the right side (70%, *p* < 0.001, Table 1**)** than the sporadic group, which could be indicative of the typical presentation of LS-related CRC. Tumor MSI-H status revealed a significantly greater proportion (67%, *p* < 0.001) of MSI-H tumors within the likely-LS group than in the sporadic group, consistent with the known association of MSI-H with LS. Interestingly, a high percentage (91.74%, *p* < 0.001) of negative BRAF-V600E protein change was observed in the sporadic CRC group, suggesting unique genetic alterations for further investigation. Moreover, the presence of significant differences between likely-LS and sporadic CRC in the presence of *MLH1, MSH2*, *PMS2, EPCAM*, and *MSH6* variants underscore the distinctive molecular profiles characteristic of LS (All *p* < 0.001, Table 1).

**Table 1:** A comparison of demographic and clinical features with phenotypes resembling likely-LS and sporadic CRC.

Characteristic	Overall (*n* = 524)	Likely-LS (*n* = 40)	Sporadic (*n* = 484)	*p*-value
**Age**				
<60 years	158 (30.15%)	13 (32.50%)	145 (29.96%)	0.7364
≥60 years	366 (69.85%)	27 (67.50%)	339 (70.04%)	
**Tumor stage**				
I - II	292 (55.73%)	28 (70.00%)	264 (54.55%)	**0.0311**
III - IV	220 (41.98%)	10 (25.00%)	210 (43.39%)	
Unknown	12 (2.29%)	2 (5.00%)	10 (2.07%)	
**Race**				
Caucasian	273 (52.10%)	17 (42.50%)	256 (52.89%)	0.3153
Non-Caucasian	73 (13.93%)	7 (17.50%)	66 (13.64%)	
Unknown	178 (33.97%)	16 (40.00%)	162 (33.47%)	
**Sex**				
Female	253 (48.28%)	19 (47.50%)	234 (48.35%)	0.9179
Male	271 (51.72%)	21 (52.50%)	250 (51.65%)	
**History of Other Malignancy**				
No	328 (62.60%)	24 (60.00%)	304 (62.81%)	0.6353
Yes	18 (3.44%)	2 (5.00%)	16 (3.31%)	
Unknown	178 (33.97%)	14 (35.00%)	164 (33.88%)	
**History of Colon Polyps**				
No	215 (41.03%)	16 (40.00%)	199 (41.12%)	0.954
Yes	108 (20.61%)	9 (22.50%)	99 (20.45%)	
Unknown	201 (38.36%)	15 (37.50%)	186 (38.43%)	
**Family History of CRC**				
0	275 (52.48%)	20 (50.00%)	255 (52.69%)	0.452
1 - 2	48 (9.16%)	5 (12.50%)	43 (8.88%)	
Unknown	201 (38.36%)	15 (37.50%)	186 (38.43%)	
**Tumour Lymphocytic Infiltration**				
High	86 (16.41%)	8 (20.00%)	78 (16.12%)	0.2542
Low	132 (25.19%)	7 (17.50%)	125 (25.83%)	
Unknown	306 (58.40%)	25 (62.50%)	281 (58.06%)	
**Tumour side**				
Left-sided	149 (28.44%)	3 (7.50%)	146 (30.17%)	**<0.001**
Right-sided	222 (42.37%)	28 (70.00%)	194 (40.08%)	
Unknown	153 (29.20%)	9 (22.50%)	144 (29.75%)	
**MSI status**				
MSS	436 (83.21%)	13 (32.50%)	423 (87.40%)	**<0.001**
MSI-H	88 (16.79%)	27 (67.50%)	61 (12.60%)	
***KRAS*** **variant**				
Negative	310 (59.16%)	25 (62.50%)	285 (58.88%)	0.649
Positive	214 (40.84%)	15 (37.50%)	199 (41.12%)	
***BRAF*** **variant**				
Negative	462 (88.17%)	24 (60.00%)	438 (90.50%)	**<0.001**
Positive	62 (11.83%)	16 (40.00%)	46 (9.50%)	
***BRAF*** **protein change**				
V600E negative	473 (90.27%)	29 (72.50%)	444 (91.74%)	**<0.001**
V600E positive	51 (9.73%)	11 (27.50%)	40 (8.26%)	
***MLH1*** **variant**				
Negative	502 (95.80%)	34 (85.00%)	468 (96.69%)	**<0.001**
Positive	22 (4.20%)	6 (15.00%)	16 (3.31%)	
***MSH2*** **variant**				
Negative	503 (95.99%)	19 (47.50%)	484 (100%)	**<0.001**
Positive	21 (4.01%)	21 (52.50%)	0 (0.00%)	
***PMS2*** **variant**				
Negative	510 (97.33%)	26 (65.00%)	484 (100%)	**<0.001**
Positive	14 (2.67%)	14 (35.00%)	0 (0.00%)	
***EPCAM*** **variant**				
Negative	521 (99.43%)	37 (92.50%)	484 (100%)	**<0.001**
Positive	3 (0.57%)	3 (7.50%)	0 (0.00%)	
***MSH6*** **variant**				
Negative	500 (95.42%)	16 (40.00%)	484 (100%)	**<0.001**
Positive	24 (4.58%)	24 (60.00%)	0 (0.00%)	

### Comparison of microsatellite status with different age categories

Different genetic markers show pronounced differences with *p-*values < 0.001(Table 2), suggesting a strong correlation between age at CRC diagnosis, tumor location, tumor mutation count. Patients with high MSI (MSI-H) tend to be diagnosed with CRC at a younger age, which is consistent with the early-age onset often observed in LS. Tumors in MSI-H patients are predominantly located on the right side of the colon and exhibit a higher mutation count, underscoring the established association between MSI-H status and a hypermutated phenotype. Additionally, the pathogenic mutations in *KRAS* and *BRAF* genes vary significantly between the MSI groups, with *p*-values of 0.0034 and <0.001, respectively, indicating a potential differential impact on the molecular pathways involved in tumorigenesis. The absence of *BRAF* V600E mutations in MSI-H patients further accentuates the distinct genetic profile this group. MMR gene mutations are notably prevalent in the MSI-H group, with *p* < 0.001, reinforcing their role in the aetiology of MSI-H colorectal cancer. These significant findings highlight the potential for tailored diagnostic and therapeutic approaches based on MSI status in CRC management. Finally, while most patients in both groups were *BRAF* V600E variant negative, a small percentage in each group were positive for pathogenic *BRAF* mutations. This finding is significant, as *BRAF* mutations can have a prognostic value in CRC and may be inversely associated with likely-LS. The two age groups within our CRC patient cohort indicate a high degree of genetic heterogeneity and a complex interplay of various clinical attributes, and molecular markers. This complexity underscores the need for the integrative machine learning scoring model being proposed, which could significantly enhance the differentiation of likely-LS patients within such a varied population (Table 2).

**Table 2:** Distribution of genetic markers and clinical features across CRC patients stratified by MSI status and age group.

	Age < 60		Age ≥ 60		All Ages		
Variable	MSI (*N* = 21)	MSS (*N* = 137)	MSI (*N* = 67)	MSS (*N* = 299)	MSI (*N* = 88)	MSS (*N* = 436)	*P*-value
**Race**							0.0286
Not known	3 (14.3%)	16 (11.7%)	19 (28.4%)	83 (27.8%)	22 (25.0%)	99 (22.7%)	
American or Alaska native	0 (0%)	0 (0%)	0 (0%)	1 (0.3%)	0 (0%)	1 (0.2%)	
Asian	0 (0%)	7 (5.1%)	1 (1.5%)	3 (1.0%)	1 (1.1%)	10 (2.3%)	
Black or African American	3 (14.3%)	22 (16.1%)	7 (10.4%)	23 (7.7%)	10 (11.4%)	45 (10.3%)	
White	11 (52.4%)	47 (34.3%)	36 (53.7%)	105 (35.1%)	47 (53.4%)	152 (34.9%)	
Missing	4 (19.0%)	45 (32.8%)	4 (6.0%)	84 (28.1%)	8 (9.1%)	129 (29.6%)	
**Sex**							**0.0167**
Female	11 (52.4%)	77 (56.2%)	39 (58.2%)	126 (42.1%)	50 (56.8%)	203 (46.6%)	
Male	10 (47.6%)	60 (43.8%)	28 (41.8%)	173 (57.9%)	38 (43.2%)	233 (53.4%)	
**Family History of CRC**							0.171
No	7 (33.3%)	62 (45.3%)	50 (74.6%)	156 (52.2%)	57 (64.8%)	218 (50.0%)	
Yes	5 (23.8%)	11 (8.0%)	6 (9.0%)	26 (8.7%)	11 (12.5%)	37 (8.5%)	
Missing	9 (42.9%)	64 (46.7%)	11 (16.4%)	117 (39.1%)	20 (22.7%)	181 (41.5%)	
**Tumour location**							**<0.001**
Left-sided	2 (9.5%)	47 (34.3%)	5 (7.5%)	95 (31.8%)	7 (8.0%)	142 (32.6%)	
Right-sided	10 (47.6%)	33 (24.1%)	48 (71.6%)	97 (32.4%)	58 (65.9%)	130 (29.8%)	
Transverse Colon	3 (14.3%)	10 (7.3%)	1 (1.5%)	15 (5.0%)	5 (5.7%)	25 (5.7%)	
Missing	6 (28.6%)	47 (34.3%)	8 (11.9%)	92 (30.8%)	14 (15.9%)	139 (31.9%)	
**Tumour Mutation Count**							**<0.001**
Mean (SD)	1470 (1570)	371 (1550)	1170 (880)	162 (528)	1240 (1080)	228 (978)	
Median [Min, Max]	1050 [510, 7490]	84.0 [12.0, 11400]	1090 [10.0, 4880]	109 [10.0, 6310]	1090 [10.0, 7490]	97.0 [10.0, 11400]	
**Pathologic Diagnosis**							**<0.001**
Colon Adenocarcinoma	12 (57.1%)	79 (57.7%)	47 (70.1%)	194 (64.9%)	59 (67.0%)	273 (62.6%)	
Colon Adenocarcinoma, Mucinous Type	5 (23.8%)	13 (9.5%)	16 (23.9%)	21 (7.0%)	21 (23.9%)	34 (7.8%)	
Rectal Adenocarcinoma	2 (9.5%)	32 (23.8%)	2 (3.0%)	34 (11.4%)	4 (4.5%)	66 (15.1%)	
Rectal Adenocarcinoma, Mucinous Type	2 (9.5%)	5 (3.6%)	2 (3.0%)	1 (0.3%)	4 (4.5%)	6 (1.4%)	
Missing	0 (0%)	4 (2.9%)	0 (0%)	5 (1.7%)	0 (0%)	9 (2.1%)	
**Microsatellite stability status**							**<0.001**
MSI	21 (100%)	0 (0%)	67 (100%)	0 (0%)	88 (100%)	0 (0%)	
MSS	0 (0%)	137 (100%)	0 (0%)	299 (100%)	0 (0%)	436 (100%)	
**KRAS variant(s)**							**0.0304**
KRAS variant negative	10 (47.6%)	93 (67.9%)	46 (68.7%)	161 (53.8%)	56 (63.6%)	254 (58.3%)	
KRAS variant positive	11 (52.4%)	44 (32.1%)	21 (31.3%)	138 (46.2%)	32 (36.4%)	182 (41.7%)	
**Pathogenic KRAS mutation(s)**							**0.0347**
KRAS mutation negative	11 (52.4%)	93 (67.9%)	46 (68.7%)	161 (53.8%)	56 (63.6%)	254 (58.3%)	
KRAS mutation positive	10 (47.6%)	44 (32.1%)	21 (31.3%)	138 (46.2%)	32 (36.4%)	182 (41.7%)	
**Variant in one or more MMR genes**							**<0.001**
MMR variant negative	15 (71.4%)	133 (97.1%)	41 (61.2%)	285 (95.3%)	50 (56.8%)	418 (95.9%)	
MMR variant positive	6 (28.6%)	4 (2.9%)	26 (38.8%)	14 (4.7%)	38 (43.2%)	18 (4.1%)	
**Pathogenic mutation in one or more MMR genes**							**<0.001**
MMR mutation negative	15 (71.4%)	133 (97.1%)	41 (61.2%)	285 (95.3%)	50 (56.8%)	418 (95.9%)	
MMR mutation positive	6 (28.6%)	4 (2.9%)	15 (22.4%)	11 (3.7%)	21 (23.9%)	15 (3.4%)	
***BRAF*** **variant**							**<0.001**
*BRAF* variant negative	19 (90.5%)	128 (93.4%)	28 (41.8%)	287 (96.0%)	47 (53.4%)	415 (95.2%)	
*BRAF* variant positive	2 (9.5%)	9 (6.6%)	39 (58.2%)	12 (4.0%)	41 (46.6%)	21 (4.8%)	
***BRAF*** **V600E mutation**							
*BRAF* mutation negative	20 (95.2%)	131 (95.6%)	29 (43.3%)	287 (96.0%)	49 (55.7%)	418 (95.9%)	**<0.001**
*BRAF* mutation positive	1 (4.8%)	6 (4.4%)	38 (56.7%)	12 (4.0%)	39 (44.3%)	18 (4.1%)	

### Genomics and mutational landscape in TP53, APC, and KRAS genes in the study cohort

**Fig. 2:**
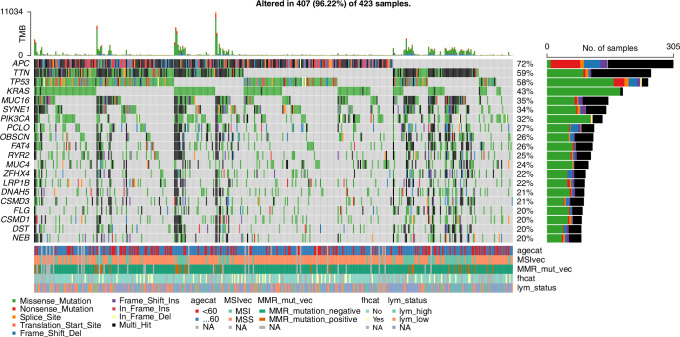
An Oncoprint illustrates the distribution of mutations across the most frequently altered genes, including *APC*, *TP53*, *KRAS*, *MUC16*, and *PIK3CA* Each column represents a single tumor sample, with different mutation types (e.g., missense mutations, nonsense mutations, frame shifts) color-coded as indicated in the legend. The bar chart on the top shows the tumor mutational burden (TMB) per sample, while the bar chart on the right summarizes the percentage of samples harbouring mutations in each gene. Clinical annotations, including age category (agecat), microsatellite instability status (MSIvec), mismatch repair (MMR) mutation status (MMR_mut_vec), immune infiltration (lym_status), and functional category (fhcat), are represented by the color bars at the bottom. High mutation frequencies and clinical correlations suggest these genes and annotations as critical factors in the genetic and immune landscape of the CRC tumors.

In a comprehensive genomic analysis, an extensive mutational burden was unveiled, with 95.22% exhibiting genetic alterations, signifying a high mutation prevalence. The oncoprint (Fig. 2) revealed a remarkable 95.22% of the cohort exhibited genetic alterations, with a conspicuous aggregation of mutations in cardinal oncogenes such as *TP53*, *APC*, and *KRAS*, suggesting these may play a central role in the oncogenic processes within the cohort. The exploration of genomic data in our cohort has yielded a comprehensive mutational framework. This mutation prevalence underscores the potential for shared oncogenic pathways and heightens the relevance of constructing a robust machine learning scoring model to discern likely-LS patients effectively. The elevated incidence of MSI-H status across the samples aligns with the immunotherapeutic axis, suggesting these patients might benefit from such interventions, a hypothesis that our machine learning model can refine and validate. The presence of a high tumor mutation burden in a subset of samples further accentuates the utility of the model, as it could potentially predict heightened immunogenicity and response to immunotherapies. Crucially, Fig. 2 illustrates not only the mutation frequency but also the co-occurrence and exclusivity patterns, providing a nuanced understanding of the genomic interplay. This complexity is captured by our integrative model, which is designed to sift through these patterns to identify those characteristic of likely LS. Demographic and clinical correlations with genomic alterations are particularly telling, signifying the potential of the model to integrate clinical phenotypes with genotypic data to enhance the accuracy of likely-LS identification. The histogram quantification of mutations per sample enriches the dataset of the model, potentially correlating extensive genomic alterations with aggressive tumor phenotypes and informing the scoring criteria of the model.

### Machine learning modelling

Of the 524 observations, 80% were randomly sampled (*n* = 420) for training the statistical models (training dataset) while the remaining 20% (*n* = 104) were used for testing the performance of the statistical models (test dataset). The data were randomly sampled according to the outcome of LS-like syndrome to preserve the original distribution of the outcome in the training and testing datasets (Table 3).

**Table 3:** Summary of datasets used in the analysis.

Dataset	Total number of observations	Likely-LS, *n* (%)	Non-LS, *n* (%)
Training	420	32 (8)	388 (92)
Test	104	8 (8)	96 (92)

Group regularisation methods in combination with 10-fold cross-validation were performed for feature selection on the training data. Three logistic regression models were developed: (1) logistic regression combined with elastic net regularisation trained using only clinical characteristics, (2) logistic regression combined with elastic net regularisation trained using clinical characteristics and MSI status, and (3) logistic regression combined with elastic net regularisation trained using both clinical and genetic characteristics.

Model fit of the four models to the training and test datasets was assessed using the classification accuracy, sensitivity, specificity, positive predictive value and negative predictive value with a classification threshold of 0.08. The performance and discriminative ability of the models were assessed using receiver operating characteristic (ROC) curves and precision-recall (PR) curves. The predictive accuracy was measured using the area under the ROC curve (AUC) and area under the PR curve (AUCPR) (Table 4).

**Table 4:** Comparison of classification accuracy measures between models used to predict LS-like syndrome.

Model and dataset	Accuracy	Sensitivity	Specificity	PPV	NVP	F1	AUC	AUCPR
Clinical (training)	0.58	0.88	0.55	0.14	0.98	0.24	0.74	0.21
Clinical (testing)	0.51	0.50	0.51	0.08	0.92	0.14	0.61	0.19
Clinical + MSI (training)	0.82	0.78	0.82	0.27	0.98	0.40	0.86	0.44
Clinical + MSI (testing)	0.83	0.75	0.83	0.27	0.98	0.40	0.83	0.32
Clinical + genetic (training)	1	1	1	1	1	1	1	1
Clinical + genetic (testing)	1	1	1	1	1	1	1	1

**Fig. 3:**
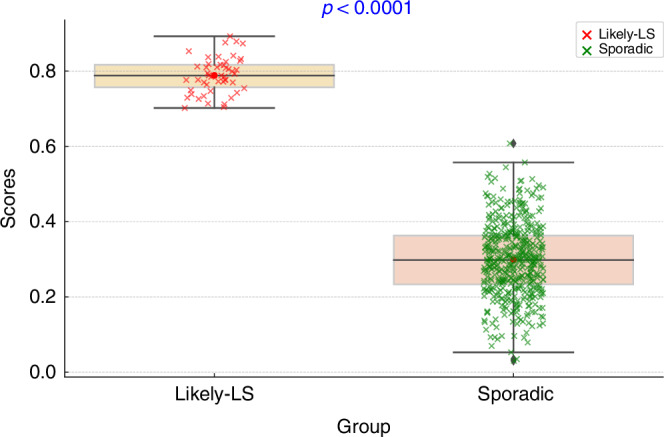
Distribution of the model-predicted scores for “Likely-LS” and “Sporadic” groups. The box plots depict the central tendency and variability of scores for each group, with individual data points overlayed for detailed visualization. The “Likely-LS” group exhibits significantly higher scores compared to the “Sporadic” group (*p* < 0.0001), as determined by a Mann-Whitney U test.

The distribution of model-predicted scores for the “Likely-LS” and “Sporadic” groups, showcasing the model’s ability to distinguish between these populations. The “Likely-LS” group shows consistently higher scores compared to the “Sporadic” group, with a clear separation in distributions while the figure supports the model’s efficacy in stratifying patients based on likelihood of LS (*p* < 0.0001, Fig. 3).

### Model performance and internal validation

**Fig. 4:**
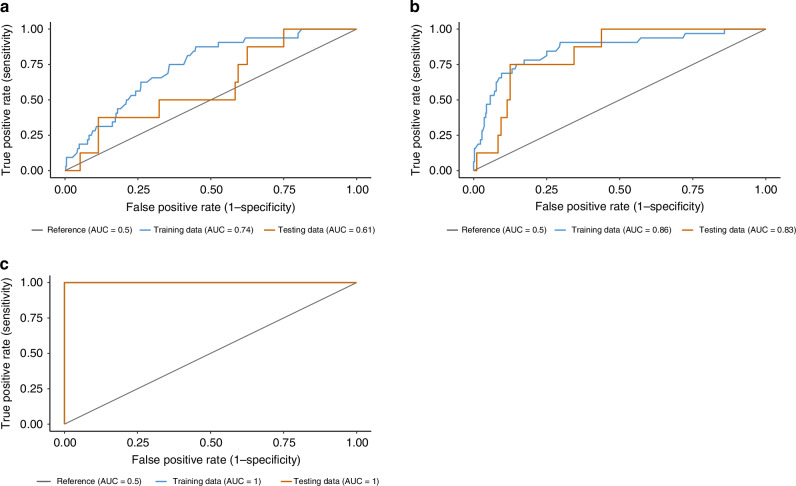
ROC curves for the model accuracy. Subplots **a** and **b** exhibit promising AUC values on training and test data, affirming the model’s discriminatory capacity, while Subplot **c**, showing perfect AUC values of 1, may indicate overfitting or an exemplary scenario in controlled conditions. This figure underscores our commitment to developing a robust, reliable screening tool, and sets the stage for a discussion on the nuances of model validation and clinical applicability.

As we endeavor to enhance the precision of LS detection, the ROC curves featured herein offer a transparent evaluation of the performance of our diagnostic model. These curves plot the true positive rate (sensitivity) against the false positive rate (1-specificity) across various threshold settings, providing a comprehensive view of the trade-offs between detecting LS cases accurately and minimizing false alarms. The AUC values serve as a quantitative measure of the overall ability of the model to discriminate between LS and non-LS patients, with the ideal model approaching an AUC of 1 (Fig. 4).

**Fig. 5:**
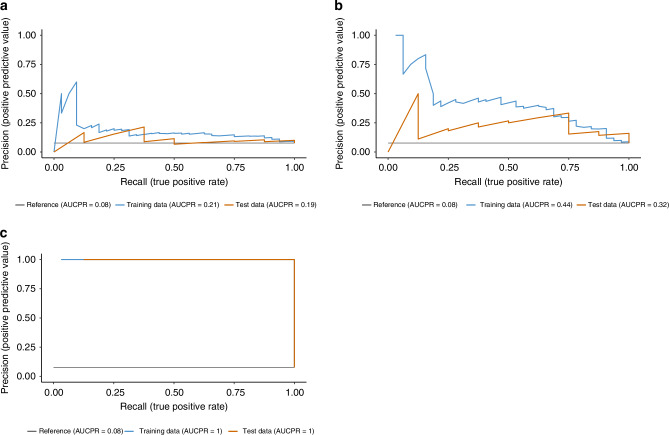
Precision-Recall Curves for LS machine learning model. Subplots **a**, **b**, and **c** illustrate the precision and recall of the model on training and test data, with AUCPR scores indicating its ability to correctly identify LS cases from a cohort of CRC patients. Subplot C shows a perfect precision of our model.

In the landscape of LS screening, where the prevalence of the condition may be low relative to the general population, the utility of Precision-Recall (PR) curves becomes particularly pronounced. PR curves are a preferred metric over traditional ROC curves in such imbalanced datasets because they focus on the performance of the classifier on the positive (minority) class, i.e. the class of interest (Fig. 5).

**Fig. 6:**
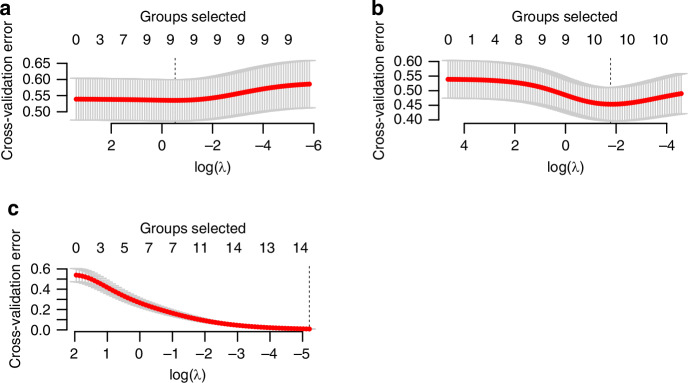
Elastic Net cross-validation plots showing the relationship between model error and regularization strength (log(λ)) for different predictor sets. **a** Clinical-only model, (**b**) Clinical + MSI model, (**c**) Full model (Clinical + Genomics). Elastic Net, used for variable selection and regularization to enhance the prediction accuracy and interpretability of our statistical model. Each subplot (**a**, **b**, and **c**) represents cross-validation error across a range of logarithmic values of the regularization parameter λ (lambda). The ‘Groups selected’ refers to the number of variables or groups of variables that the model includes at each λ value. **a** Demonstrates a relatively stable selection of groups and cross-validation error across a wide range of λ values, suggesting that the model is robust to changes in the regularization parameter.

For cross-validation error against different levels of regularization strength, informing our choice of the optimal number of predictive features to include, we performed analyses which are crucial for refining a model that is both accurate and generalizable, ensuring it captures the essential predictors for LS without succumbing to overfitting or unnecessary complexity. The Elastic Net regularization technique is an advanced method that combines the penalties of both the Lasso (L1 regularization) and Ridge (L2 regularization) methods. It is particularly well-suited for situations where there are multiple features that may be correlated or when the number of predictors is greater than the number of observations (Fig. 6). Incorporating Elastic Net into our study represents a strategic choice to build a robust and interpretable model that can leverage the rich genomic and clinical data available for effective LS screening.

In the context of LS screening, these plots would be used to identify the most parsimonious model that adequately captures the complexity of the data without overfitting, potentially improving the generalizability and predictive accuracy of the model when applied to new patient data.

### Risk score bins for likely-LS

The risk score bins for likely-LS with corresponding probability ranges based on different assessment criteria is shown in Table 5. The Clinical and genetic risk score column shows a substantial jump in the probability range for likely-LS, particularly noticeable in the 11–20 risk score bin where the upper range dramatically increases to 35.9%, and from the 21–30 risk score bin onward, the probability exceeds 90%, indicating a high likelihood of LS when genetic factors are accounted for (Table 5).

**Table 5:** Risk score bins with corresponding probability of likely-LS.

Risk score bins	Clinical risk score probability of likely-LS range	Clinical + MSI risk score probability of likely-LS range	Clinical + genetic risk score probability of likely-LS range
0 –10	4.8–5.7	1.5–2.8	0.1–2.3
11–20	5.8–6.8	2.9–5.0	2.4–35.9
21–30	6.9–8.1	5.1–8.8	36.0–92.9
31–40	8.2–9.6	8.9–14.8	93.0–99.7
41–50	9.7–11.3	14.9–23.8	99.7–100
51–60	11.4–13.2	23.9–36.0	99.9–100
61–70	13.3–15.4	36.1–50.4	99.9–100
71–80	-	50.5–64.6	99.9–100
81–90	-	-	99.9–100
91–99	-	-	99.9–100

This table highlights the importance of integrating genetic data with clinical and MSI factors to improve the accuracy of risk assessment tool for LS-like syndrome. The substantial increase in probability ranges with the inclusion of genetic data underscores the significance of the genetic component in LS risk prediction in our machine learning model.

Our model significantly outperforms these approaches in terms of sensitivity and specificity, driven by its ability to incorporate both genomic and molecular data, including somatic mutation profiling from large datasets like TCGA. Several well-established models for predicting LS have been developed, such as PREMM5, MMRpro, and the Bethesda guidelines. However, these models rely primarily on clinical features and family history, which limits their accuracy in certain populations. Unlike these traditional models, we have compared our machine learning approach which integrates somatic mutation data with clinicopathological features, offering a more comprehensive and accurate prediction of likely-LS cases (Table 6). PREMM5: This model predicts the likelihood of identifying a germline mutation in one of the LS-associated genes (*MLH1, MSH2, MSH6, PMS2*) and *EPCAM*) using personal and family history. However, it does not incorporate somatic mutations or molecular features like MSI status. MMRpro: Like PREMM5, MMRpro uses family history and tumor characteristics but lacks integration of genomic data, such as somatic mutations. Bethesda guidelines: The Bethesda guidelines are widely used to determine which CRC patients should undergo further testing for LS. However, their reliance on clinical criteria alone makes them less precise than models that integrate molecular and genomic data.

**Table 6:** Performance metrics with other LS prediction models.

Model	Data Used	Sensitivity	Specificity	AUC	Somatic Mutation Integration	MSI Status
Our Model	Clinicopathological + Genomics	100%	100%	1	Yes	Yes
PREMM5	Clinical + Family History	88%	72%	0.86	No	No
MMRpro	Clinical + Family History	90%	65%	0.85	No	No
Bethesda Guidelines	Clinical Criteria	81%	58%	0.75	No	No

## Discussion

We present a novel machine-learning scoring model approach that significantly advances the ascertainment of likely-LS cases within a large cohort of CRC patients in the cBioPortal. The novelty of our study lies in its multifaceted approach using clinical and somatic mutations to distinguishing likely-LS within the broader spectrum of a CRC cohort, a task that has historically presented a diagnostic conundrum due to its phenotypic mimicry of LS. The unparalleled sensitivity, specificity, and accuracy of the model with AUC values reaching the ideal score of 1(100%), delineate a new frontier in the predictive diagnosis of LS. Our results underscore the transformative potential of integrating comprehensive genomic and clinicopathological data, leveraging advanced machine learning techniques to enhance the precision of LS ascertainment. By integrating clinicopathological, MSI, and extensive genomic data, the model achieves a perfect sensitivity, specificity, and accuracy, demonstrating a quantum leap over existing screening methodologies. Our research advances the field by integrating somatic genomics and clinical data into a machine learning predictive model to discriminate likely-LS patients from a cohort of CRC patients, an approach not previously employed in LS research. It is a quintessential example of how machine learning can be leveraged to streamline complex decision-making processes in clinical settings, potentially applying translational research to precision medicine especially in resource-limited setting.

Our comprehensive data integration strategy transcends traditional diagnostic paradigms, capturing the intricate interplay between genetic, clinical, and MSI-related factors associated with LS. This holistic approach not only enhances the predictive power of the model but also underscores the complex aetiology of LS, offering insights into its underlying mechanisms. The translational potential of our findings can be exemplified by the development of an AI-powered LS ascertainment web tool. This tool can embody the practical application of our model formula, offering a rapid, accurate, and accessible means for LS screening in limited resource settings for germline testing. Its implementation could revolutionize genetic diagnostics and personalized healthcare strategies, particularly in resource-limited settings, aligning with the goals of precision medicine. Moreover, our comprehensive approach to data integration encompassing clinicopathological, MSI, and genetic data, encapsulates the essence of holistic patient assessment. This multifaceted model not only captures the complex interplay of factors associated with LS but also paves the way for a more nuanced understanding of CRC and its hereditary components. By offering near-perfect predictive performance, the model promises to revolutionize LS screening protocols, making them more efficient, accessible, and cost-effective, particularly in resource-limited settings. This aligns seamlessly with the goals of personalized medicine, enabling targeted interventions and tailored patient care.

Our study revealed a significant absence of *BRAF* V600E mutations in microsatellite instability-high (MSI-H) cases, and a predominance of mutations in pivotal oncogenes such as *TP53, APC, and KRAS* which offers novel insights into the genetic underpinnings of LS. However, in our machine learning models, features such as *TP53*, *APC*, and *KRAS* mutations were excluded after feature selection due to their redundancy with other highly predictive markers, such as MSI and MMR gene mutations. While these mutations are pivotal in CRC pathogenesis, their lower specificity for LS limited their contribution to the model’s predictive accuracy. Furthermore, the presented mutational landscape provides a valuable genomic signature that can refine molecular criteria for LS diagnosis, potentially guiding targeted therapeutic interventions. The predictive performance of our machine learning model built upon these genomic and clinical foundations, underscores a profound scientific opportunity: (i) to dissect the complex aetiological landscape of LS and, (ii) harness this understanding for the advancement of precision medicine in CRC genetics. Adoption of Elastic Net regularization in our model underscores a methodologically rigorous approach, balancing the L1 and L2 penalties to effectively manage multicollinearity and feature selection. This methodological choice not only mitigates the risk of overfitting but also augments the interpretability and generalizability of the model, ensuring its applicability across diverse clinical settings. Furthermore, the study highlights the critical role of combining genomic data in LS ascertainment.

By unveiling a demographic bias towards younger, non-Caucasian individuals within the likely-LS cohort, our findings challenge the prevailing understanding of LS epidemiology, suggesting that likely-LS may be more prevalent and phenotypically distinct in diverse populations than previously recognized. Moreover, the identification of a robust correlation between the presence of key oncogenic mutations and likely-LS status provides a novel genomic signature that could aid in the early detection of LS. Our machine learning model’s capability to integrate these complex datasets into a predictive tool is groundbreaking, promising a significant leap forward in the personalized screening for LS primarily using clinical and somatic data (likely in the near future) for cascade testing. This leap has substantial scientific impact, heralding a new era in the ascertainment of likely-LS, by extending the precision of germline testing to confirm LS diagnosis [77].

The internal validation of our model, as evidenced by the ROC and Precision-Recall curves, underscores its reliability and efficacy. Such a model, with near-perfect predictive performance, represents a significant leap forward in the personalized screening and management of LS, offering a beacon of hope for patients and a new paradigm for clinicians and researchers. The discovery of LS amidst CRC patients presents an intricate challenge due to its complex diagnostic approach and consequential implications for targeted interventions. However, the present study elucidates the demographic, clinical, and genomic landscapes of a cohort of CRC patients, unraveling nuanced patterns indicative of likely-LS. The predominance of early-onset CRC in patients under 60 years with MSI-H, and absence of *BRAF*-V600E mutations, coupled with a higher incidence of early-stage tumors, reinforces the known hypothesis that LS manifests earlier than sporadic CRC. In addition, this was corroborated by the high tumor-infiltrating lymphocytes (TILs) in the likely-LS group, suggesting an immunogenic profile characteristic of LS [34]. The significant absence of *BRAF* V600E mutations among MSI-H patients further distinguishes likely-LS from sporadic MSI-H CRC, which is typically associated with *BRAF* V600E mutations [78, 79]. This underscores the necessity for refined diagnostic criteria that capture the unique genetic fingerprint of likely-LS, which may diverge from classical LS mutations. The comprehensive genomic analysis reaffirmed the prevalence of mutations in pivotal oncogenes (*TP53, APC, KRAS*) highlighting the mutational burden that defines CRC.

The application of Elastic Net regularization stands out as a methodological keystone, ensuring a balanced consideration of model complexity and feature relevance. This approach mitigates the risk of overfitting, a common pitfall in machine learning applications, thereby enhancing the generalizability of the model across diverse clinical settings. Such methodological rigor is imperative in the high-stakes arena of cancer diagnostics, where the balance between sensitivity and specificity can directly influence patient outcomes and treatment pathways.

This intricate interplay of genetic, clinical, and demographic factors in CRC underscores the complexity of personalized medicine. Our machine learning model, trained on this genomic and clinical data, has shown exceptional promise, achieving near-perfect predictive accuracy. Such precision, evidenced by the robust Area Under the Curve (AUC) values of the model, heralds a new epoch in the precision screening of LS. The utility of our model transcends conventional risk assessment, offering a precise, quantifiable probability of likely-LS across a spectrum of risk score bins (Table 3). This risk scoring, derived from Elastic Net regularization, embodies a significant stride toward personalized medicine, enabling clinicians to pinpoint high-risk individuals for targeted genetic testing and surveillance. The integration of genetic markers with clinical and MSI data exemplifies the capacity of the model to discern complex patterns, a feat unattainable by traditional statistical methods.

Validation of our approach in diverse populations will be imperative to ensure the generalizability and efficacy of the model across different genetic backgrounds. The precision of our automated risk scoring system, integrating clinical, MSI, and genetic data, offers a quantifiable measure of LS risk. This granular risk stratification facilitates the deployment of targeted surveillance and preventive measures, aligning with the overarching goals of precision oncology. Moreover, the employment of elastic net regularization emphasizes our commitment to methodological rigor, ensuring that the selected features are truly predictive of likely-LS while mitigating the risk of model overfitting. This findings of the study impel us to consider the broader implications for cancer diagnostics and the burgeoning field of precision medicine. The integration of machine learning with multi-dimensional clinical and genomic data sets a new benchmark for diagnostic models, offering a paradigm that is both scalable and adaptable to other hereditary cancer syndromes. Future studies should aim to validate the model across various populations and healthcare settings to ensure its generalizability and effectiveness. Moreover, the integration of additional genomic markers and other omics data could further refine the predictive capabilities of the model, providing even deeper insights into LS and CRC.

The predictive prowess of our machine learning models, as evidenced by the high AUC values, is indicative of their potential in clinical applications. However, the perfect predictive accuracy observed in the clinical-genetic model on the test dataset warrants cautious interpretation. While it may suggest a model with excellent fit, it raises concerns about overfitting, which could diminish the applicability of the model in a broader clinical context. Further external validation is necessary to confirm the performance of the model and to ensure its reliability in diverse clinical settings.

## Conclusions

This study represents a significant leap forward in the realm of precision oncology, showcasing the profound impact of integrating machine learning with comprehensive clinical data and somatic genomics data for the ascertainment of likely-LS in a cohort of CRC patients. By achieving unparalleled predictive accuracy, our model not only refines the diagnostic process for likely-LS cases but also paves the way for more personalized, efficient, and accessible approaches to CRC care. The potential development of an AI-powered web tool based on this model further underscores the translational potential of our research, offering a practical solution for clinicians and enhancing the scope of precision medicine. Ultimately, this work illuminates the path toward a future where the complex interplay of clinically available somatic genomics data, machine learning models, and clinical insights to inform every aspect of patient care, heralding a new era in the management and treatment of hereditary cancer syndromes. The implications of our work extend far beyond the confines of LS screening, serving as a template for the application of machine learning in addressing myriad challenges within oncology and beyond. By harnessing the vast potential of somatic genomics data and integrating it with clinical insights, we pave the way for personalized medicine approaches that are finely tuned to the genetic and clinical landscapes of individual patients. The future of oncology lies in the fusion of technology and medicine, and our work is a critical step towards realizing this vision. We invite the scientific community to build upon our findings, exploring new frontiers in the quest for precision medicine and ultimately transforming patient care in oncology and beyond.

## Data Availability

All data generated or analyzed during this study are included in this manuscript. The minimal dataset underlying the findings of this study, necessary for interpretation and replication, is available in the dataset file attachment, and can be accessed with the manuscript submission. For any additional data requests and inquiries should be directed to the corresponding author in accordance with relevant data privacy regulations.
